# Impact of international travel and border control measures on the global spread of the novel 2019 coronavirus outbreak

**DOI:** 10.1073/pnas.2002616117

**Published:** 2020-03-13

**Authors:** Chad R. Wells, Pratha Sah, Seyed M. Moghadas, Abhishek Pandey, Affan Shoukat, Yaning Wang, Zheng Wang, Lauren A. Meyers, Burton H. Singer, Alison P. Galvani

**Affiliations:** ^a^Center for Infectious Disease Modeling and Analysis, Yale School of Public Health, New Haven, CT 06520;; ^b^Agent-Based Modelling Laboratory, York University, Toronto, ON M3J 1P3, Canada;; ^c^State Key Laboratory of Mycology, Institute of Microbiology, Chinese Academy of Sciences, 100101 Beijing, China;; ^d^Department of Biostatistics, Yale School of Public Health, New Haven, CT 06510;; ^e^Department of Integrative Biology, The University of Texas at Austin, Austin, TX 78712;; ^f^Santa Fe Institute, Santa Fe, NM 87501;; ^g^Emerging Pathogens Institute, University of Florida, Gainesville, FL 32610

**Keywords:** COVID-19, SARS-CoV-2, surveillance, screening, disease importation

## Abstract

The novel coronavirus outbreak (COVID-19) in mainland China has rapidly spread across the globe. Within 2 mo since the outbreak was first reported on December 31, 2019, a total of 566 Severe Acute Respiratory Syndrome (SARS CoV-2) cases have been confirmed in 26 other countries. Travel restrictions and border control measures have been enforced in China and other countries to limit the spread of the outbreak. We estimate the impact of these control measures and investigate the role of the airport travel network on the global spread of the COVID-19 outbreak. Our results show that the daily risk of exporting at least a single SARS CoV-2 case from mainland China via international travel exceeded 95% on January 13, 2020. We found that 779 cases (95% CI: 632 to 967) would have been exported by February 15, 2020 without any border or travel restrictions and that the travel lockdowns enforced by the Chinese government averted 70.5% (95% CI: 68.8 to 72.0%) of these cases. In addition, during the first three and a half weeks of implementation, the travel restrictions decreased the daily rate of exportation by 81.3% (95% CI: 80.5 to 82.1%), on average. At this early stage of the epidemic, reduction in the rate of exportation could delay the importation of cases into cities unaffected by the COVID-19 outbreak, buying time to coordinate an appropriate public health response.

## Significance

To contain the global spread of the 2019 novel coronavirus epidemic (COVID-19), border control measures, such as airport screening and travel restrictions, have been implemented in several countries. Our results show that these measures likely slowed the rate of exportation from mainland China to other countries, but are insufficient to contain the global spread of COVID-19. With most cases arriving during the asymptomatic incubation period, our results suggest that rapid contact tracing is essential both within the epicenter and at importation sites to limit human-to-human transmission outside of mainland China.

In December 2019, a novel coronavirus outbreak, COVID-19, caused by the severe acute respiratory syndrome coronavirus 2 (SARS-CoV-2), emerged in Wuhan, China, as a cluster of cases exhibiting pneumonia-like symptoms (1). Within a few weeks, the outbreak spread to 24 other countries around the world, including the United States, Canada, the United Kingdom, France, Australia, and Japan (2). As of February 21, 2020, more than 76,000 cases have been confirmed globally, of which 1.8% have been reported outside mainland China (2, 3).

The current COVID-19 outbreak marks the third novel coronavirus emergence in the 21st century, after the 2003 SARS and the 2013 Middle East respiratory syndrome (MERS). The 2003 SARS epidemic originated in China’s Guangdong province, resulting in 5,327 cases within the country and 8,096 infections globally (4). In response to its global spread, many countries implemented airport screening, which was later found to be ineffective in slowing the spread of disease (5–7). As of February 21, the World Health Organization has reported 566 confirmed cases in 26 other countries, 14 of which have reported human-to-human transmission (8), with an additional 634 cases being confirmed on a cruise ship (8).

On January 23, 2020, China enacted a lockdown in Wuhan City to limit the spread of COVID-19 outbreak, which was expanded to 15 other cities in Hubei province on January 24 (9–15). With the global dissemination of SARS-CoV-2 cases out of China, several countries have instituted border measures, including symptom screening and restricting travel to and from China (16–19). Some countries are encouraging passengers from China to self-identify possible exposure via health questionnaires. Despite these interventions, the global case count continues to rise. Currently, there is limited understanding about how international flight connections and border measures are impacting the global dissemination of the COVID-19 outbreak.

We used daily COVID-19 incidence data and global airport network connectivity from mainland China to estimate country-level exportation risks of the outbreak. As expected, we found a significant correlation between the timing of global exportation events and airline connectivity with mainland China. We next evaluated the effectiveness of border measures, including travel lockdown, contact tracing at the epicenter, and airport screening in containing the spread of disease. In our analysis, we accounted for early epidemiological estimates of COVID-19 outbreak from the literature, suggesting that the average incubation period of the disease is 5.2 d but can extend to more than 12 d for some individuals (10, 20). Using Monte Carlo simulations, we estimated that about 64% of exported cases were in the presymptomatic incubation period upon arrival, indicating airport screening is unlikely to prevent disease importation on its own. We further found that the Wuhan and subsequent Hubei travel lockdowns reduced the rate of disease exportation by 81% and averted 71% cases by February 15, 2020, compared to no border restriction. Border control measures are thus unlikely to contain the outbreak, but could likely delay further importation of SARS CoV-2 cases at the early stage of the epidemic.

## Results

### Exportation Risk and Impact of Border Control Measures.

**Fig. 1.**
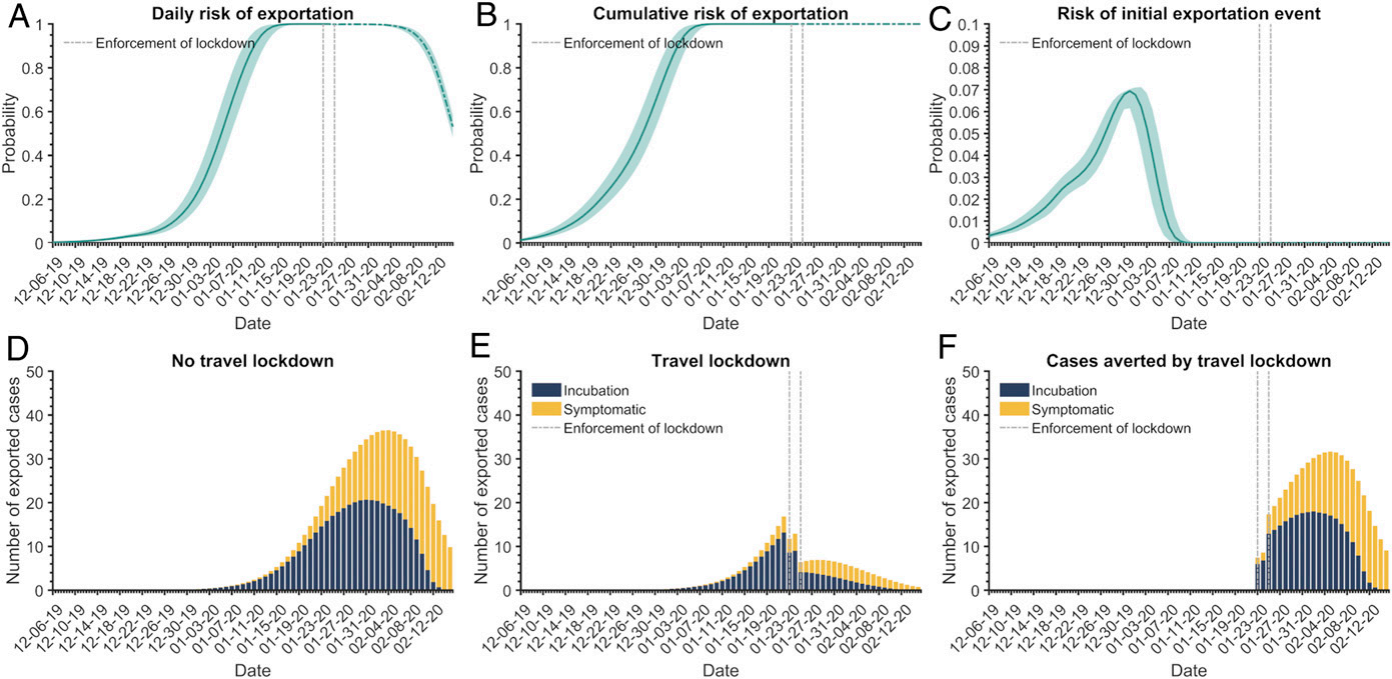
Exportation risk of SARS-CoV-2 cases from mainland China. (*A*) Daily risk, (*B*) cumulative risk of exportation of at least one case, and (*C*) risk of the initial exportation event from mainland China between December 6, 2019 and February 15, 2020. (*D* and *E*) Expected number of exported cases from mainland China traveling during the incubation period (blue) or symptomatic phase of the infection (yellow) (*D*) in the absence of travel lockdowns and (*E*) with travel lockdowns enforced. (*F*) Cases averted by the travel lockdowns. The colored band in *A*−*C* denotes the 95% credible interval. The vertical dashed lines indicate the start of two travel lockdowns in China: January 23, 2020 in Wuhan and January 25, 2020 for other cities in the Hubei province.

By January 13, 2020 (95% CI: January 11−14), the daily risk of exporting at least a single infected case from mainland China via international travel exceeded 95% (Fig. 1*A*). We estimated that the first case was exported from mainland China on December 26, 2019 (95% CI: December 24−28), with a SD of 7.2 d. We found that December 31, 2019 (95% CI: December 31, 2019 to January 2, 2020) had the greatest likelihood for the first exportation event out of China (Fig. 1*C*), corresponding to 23 d (95% CI: 21 to 23) before the initiation of the travel lockdown in Wuhan. These estimates are relatively comparable to the arrival of infected individuals in Australia and Japan on January 6, 2020 (Table 1 and *SI Appendix*, Table S5).

**Table 1.** The estimate of the date of an infected case being exported to a given country in comparison to the first arrival of a reported infected case

Country	Airline weight	Arrival date of first case	Estimated arrival date (MLE)	Estimated arrival date (mean)	SD (days)
Australia	0.00352	January 6, 2020	January 18, 2020	January 15, 2020	7.1
Japan	0.0235	January 6, 2020	January 9, 2020	January 6, 2020	6.5
Thailand	0.00743	January 8, 2020	January 14, 2020	January 11, 2020	6.5
Nepal	0.00117	January 9, 2020	January 22, 2020	January 20, 2020	8.3
Taiwan	0.027	January 12, 2020	January 8, 2020	January 5, 2020	6.6
India	0.00235	January 13, 2020	January 20, 2020	January 18, 2020	7.7
United States	0.00665	January 13, 2020	January 15, 2020	January 12, 2020	6.5
Vietnam	0.00352	January 13, 2020	January 18, 2020	January 15, 2020	7.1
United Arab Emirates	0.00235	January 16, 2020	January 20, 2020	January 18, 2020	7.7
Germany	0.00313	January 19, 2020	January 18, 2020	January 16, 2020	7.3
South Korea	0.0192	January 19, 2020	January 10, 2020	January 7, 2020	6.5
Sri Lanka	0.000391	January 19, 2020	January 22, 2020	January 23, 2020	8.6
Singapore	0.00665	January 20, 2020	January 15, 2020	January 12, 2020	6.5
Philippines	0.0043	January 21, 2020	January 17, 2020	January 14, 2020	6.9
Canada	0.00235	January 22, 2020	January 20, 2020	January 18, 2020	7.7
France	0.00117	January 22, 2020	January 22, 2020	January 20, 2020	8.3
Cambodia	0.0028	January 23, 2020	January 19, 2020	January 17, 2020	7.5
Finland	0.0008	January 23, 2020	January 22, 2020	January 22, 2020	8.5
Italy	0.0016	January 23, 2020	January 22, 2020	January 19, 2020	8.1
Sweden	0.0004	January 24, 2020	January 22, 2020	January 23, 2020	8.6
United Kingdom	0.0008	January 30, 2020	January 22, 2020	January 22, 2020	8.5

The maximum likelihood estimate (MLE) is the date of the maximum likelihood from the calculated probability distribution.

The two travel lockdowns have reduced the exportation rate from mainland China, but are insufficient to contain geographic spread (Fig. 1). Since the time of the initial lockdown on January 23, we estimate that the number of exported cases would have exceeded 50 by January 25, 2020 (95% CI: January 24−26) without border restrictions, compared to January 28, 2020 (95% CI: January 27−30) under the enforcement of the travel lockdowns. Over the course of the 24 d of the travel lockdowns, we found that an average of five cases (95% CI: 4 to 6) were exported per day, an 81.3% (95% CI: 80.5 to 82.1%) average reduction in the exportation rate predicted in the absence of any border measures. We estimate that the travel lockdowns reduced the number of COVID-19 importations by February 15, 2020, from 779 (95% CI: 632 to 967) to 230 (95% CI: 178 to 298). This is consistent with the 175 confirmed international SARS-CoV-2 infections by February 21, 2020, with travel history to China (8), taking reporting delays and an asymptomatic incubation period into account.

We estimated that 64.3% (95% CI: 55.4 to 71.3%) of exported cases were presymptomatic upon arrival at their destination airport. Assuming that only symptomatic cases were detected by airport screening upon arrival, we estimate that screening contained 82 (95% CI: 72 to 95) cases imported from mainland China, while the two travel lockdowns averted 549 (95% CI: 451 to 670) cases being exported up to February 15, 2020.

### Country-Level Importation Risk.

**Fig. 2.**
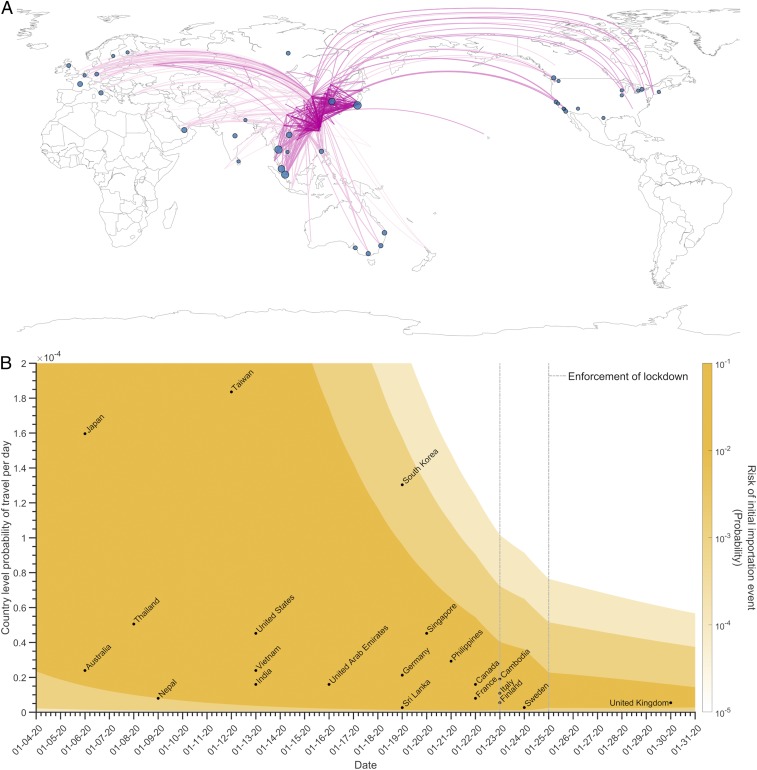
Country-level importation of SARS-CoV-2 cases. (*A*) International flight connections from mainland China. Color of the curves indicates the number of airports with flights to/from mainland China—lighter color are the routes to countries with the most airport connections. The blue circles indicate the number of international confirmed cases. Size of circles is proportional to the number of confirmed SARS-CoV-2 cases with travel history to China as of February, 15, 2020. (*B*) The risk of initial importation (yellow gradient) based on probability of an individual in mainland China to travel by flight (*y* axis), estimated each day between January 4, 2020 and January 31, 2020 (*x* axis). We found a statistically significant correlation (*r* = −0.43, *P* value = 0.05) between the reported arrival date of the initial case in 21 countries/regions and the airline weights proportional to the number of airports in the country with direct flights to and from mainland China. Vertical dashed lines indicate the travel bans that were enforced on January 23 in Wuhan and on January 25 for other cities in Hubei province.

We used the global airport network to estimate the daily probability of an initial importation event for 63 countries/regions that have direct flight connectivity to mainland China (Fig. 2). We assume the importation risk is proportional to the number of airports in the country with direct flights to and from mainland China. As expected, we found that the reported arrival of the first importations for these countries was negatively correlated with the number of airports that had direct flights to and from mainland China for 21 countries/regions as of January 30, 2020 (*r* = −0.43, *P* value = 0.05; Table 1 and Fig. 2*B*). For example, Japan, with 60 such airports, and Sri Lanka, with one such airport, reported their first COVID-19 importations on January 6 and January 19, respectively (Fig. 2*B*). As validation, our predicted first arrival times are generally consistent with reported international importation arrival dates (Table 1 and *SI Appendix*, Table S5).

### Limiting Importation through Self-Identification upon Arrival.

**Fig. 3.**
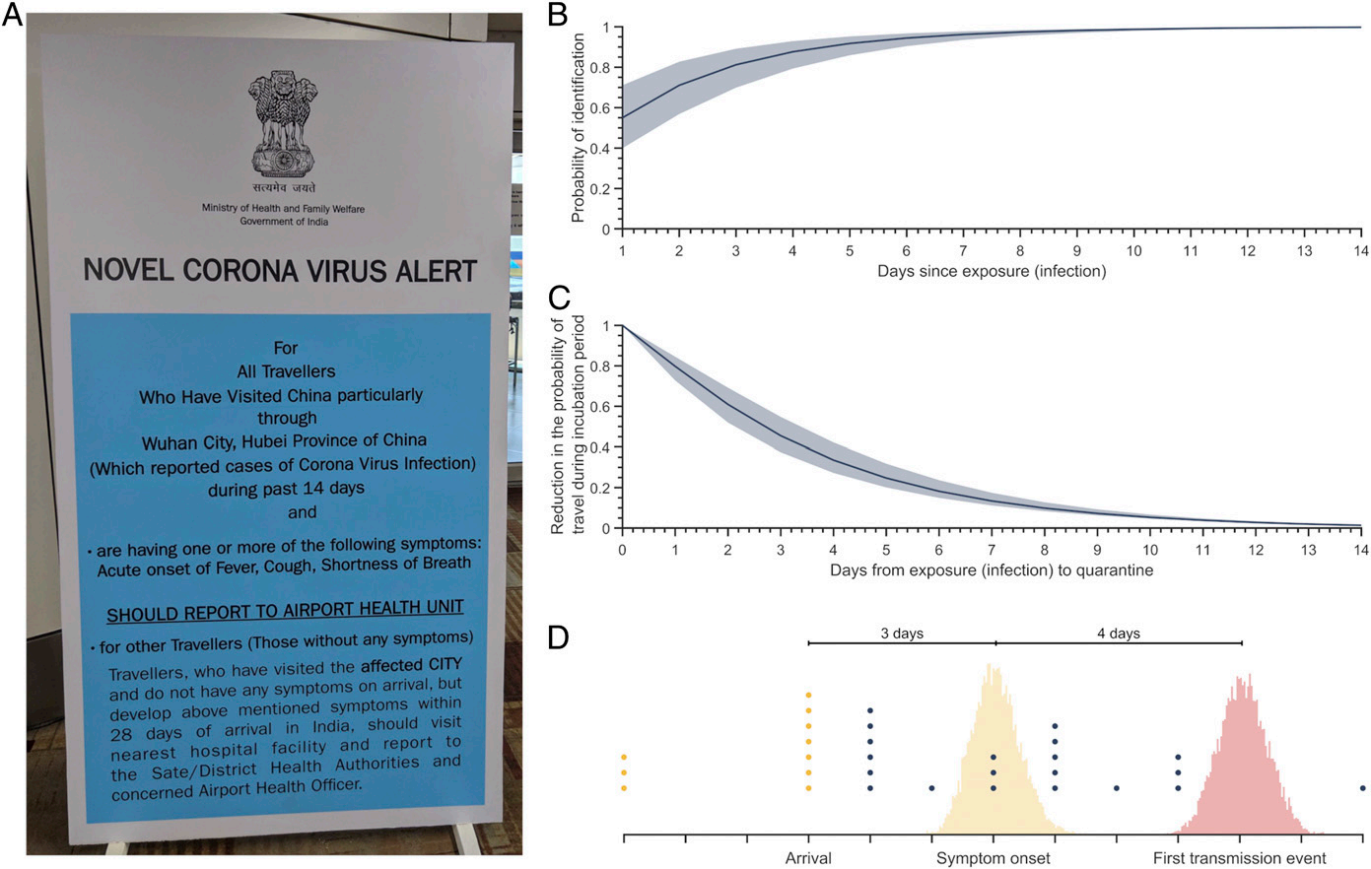
Interventions to limit the impact of infected passengers in their incubation period. (*A*) Photograph of an alert in Delhi Indira Gandhi International Airport, India, educating passengers on the proper process if they develop symptoms. (*B*) The probability of identifying an infected individual traveling during their incubation period based on asking the time since their last exposure (infection) to a COVID-19 affected area. Colored band denotes the 95% credible interval. (*C*) The probability of an infected case traveling during their incubation period for varying lags in quarantine in mainland China following exposure (infection). Colored band denotes the 95% credible interval. (*D*) Estimated time between arrival of a case in the incubation period and symptom onset (yellow area) as well as first transmission event (red area). Time of symptom onset data for 30 imported cases worldwide is shown as dots—blue dots are the cases that displayed no symptoms upon arrival, and yellow dots are cases that were symptomatic upon arrival.

Several countries have asked travelers that have visited areas affected by the COVID-19 outbreak to self-monitor for symptoms for 28 d after arrival (Fig. 3*A*). We estimated that health questionnaires for self-identification at airports inquiring about any exposure at least a week (6.3 d [95% CI: 4.9 to 7.7]) prior to arrival would catch 95% of cases traveling during the incubation period (Fig. 3*B*).

### Quarantine at the Epicenter to Curb Exportation.

We assessed the impact of quarantine (i.e., isolation of an infected individual following exposure to infection) following contact tracing in the epicenter on preventing the international travel of cases during the incubation period. The expected benefits of contact tracing depend on the rapidity with which exposed cases are identified. For example, the likelihood of an infected case traveling during the incubation period would be reduced by 24.7% (95% CI: 20.1 to 31.8%) if the case is identified via contact tracing within 5 d after exposure. By contrast, quarantining a case 10 d after exposure would only reduce the probability of travel by 5.3% (95% CI: 4.7 to 6.7%) (Fig. 3*C*).

### Local Transmission following Case Importation.

The arrival times and symptom onset of 30 reported cases outside of mainland China indicate that ∼67% of cases arrived during their incubation period (Fig. 3*D* and *SI Appendix*, Table S5). For such cases, we estimated the average duration between arrival and symptom onset is 3 d (95% CI: 2.3 to 3.9) (Fig. 3*D*). Our estimate is consistent with data from 20 importation cases around the world, indicating 3.5 d (±2.2 SD) between arrival and symptom onset (Fig. 3*D*, blue dots). Assuming the serial interval to be distributed with an average of 7.5 d and SD of 3.4 d (10), we estimated that the first transmission event occurs, on average, 4 d (95% CI: 3.2 to 4.8) after symptom onset (Fig. 3*D*). Thus, we expect the first local transmission event to typically occur within 7 d (95% CI: 6.0 to 8.2) after the arrival of an infected case in the incubation period.

### Estimation of Case Underreporting.

We estimated the probability of travel per day for any individual from mainland China to be 0.0068 (95% CI: 0.0059 to 0.0079), which was reported to be 0.005 (21). Our higher estimate compared with that previously reported underscores the likelihood of case underreporting in China, as has been widely speculated. Specifically, the relative difference in these probabilities suggests an underreporting rate of 26.5% (95% CI: 15.3 to 36.7%) from December 8, 2019 to February 15, 2020. Even under a more conservative assumption that infected people travel up to the point of hospitalization, our estimates indicate 16.7% (95% CI: 3.8 to 28.6%) underreporting.

## Discussion

Less than 2 mo after the first international reports of COVID-19 in mainland China, international flights have brought COVID-19 cases to 26 countries. As of February 21, 2020, these countries have collectively reported 556 confirmed cases (8). An additional 634 cases have been confirmed outside of mainland China on a cruise ship (8). To limit the global spread of COVID-19, symptom screening of incoming passengers has been implemented at airports in several countries. However, with early estimates suggesting a presymptomatic incubation period lasting up to 2 wk (or more), a large proportion of infected travelers may not be detected during screening.

In the calibration of our model, we considered two scenarios, for the possibility of a symptomatic individual to travel until 1) first medical visit, and 2) hospitalization (*SI Appendix*, Table S3). We found that the effectiveness of screening depends on this assumption, with longer symptomatic travel periods corresponding to a higher proportion of cases detected by February 15, 2020. If cases travel up to their first clinical visit [between 4.6 d and 5.8 d after symptom onset (10)], then we estimated that 64.3% (95% CI: 55.4 to 71.3%) of international COVID-19 case importations out of China were still presymptomatic (incubating) while traveling; if cases travel up to the point of hospitalization [between 9.1 d and 12.5 d after symptom onset (10)], this proportion reduces to 49.5% (95% CI: 40.4 to 57.4%), which is comparable to a previously published estimate of 46% (22). Additional information on both the incubation period and travel behavior of symptomatic individuals is required to provide more-accurate estimates of effectiveness of airport screening.

On January 23, a lockdown was enforced in the city of Wuhan, followed by 15 more cities in Hubei province on January 24 (9–13). We estimated that 779 (95% CI: 632 to 967) COVID-19 cases would have been exported by February 15, 2020 if these border restrictions had not been enacted. Therefore, our results indicate that the travel lockdowns enforced by the Chinese Government averted 71% of these cases. A previous analysis evaluated city-level risk of importation events between January and March (23) but did not consider importation timing. Our analysis, which utilizes incidence data in mainland China, suggests that the first exportation event was likely to have occurred almost 3 wk before the travel ban. Our prediction of exportation timings is consistent with the timing and locations of the first 21 COVID-19 cases reported outside of China.

Our results demonstrate that travel restrictions cannot be expected to fully arrest the global expansion of COVID-19, but may decrease the rate of case exportations if enacted during the early stages of the epidemic (5–7). For example, our results indicate that the travel lockdowns in Hubei decreased the rate of exportation during the early stages of the outbreak by 81%. At this early stage of the COVID-19 epidemic, this decrease in exportation rate from mainland China could delay the onset of outbreaks in cities yet to be affected. This obstruction of importation events will be critical to preparing an effective public health response for when a local COVID-19 outbreak arises.

In addition to border control measures, contact tracing is being conducted in China to identify individuals potentially exposed to SARS-CoV-2 (24–26). Our results suggest that the benefits of contact tracing depend on its rapidity. These findings highlight the importance of improving contact tracing within the epicenter in alleviating the risk of importation, as the efficacy of contact tracing in unaffected countries will largely depend on their preparedness, vigilance, and available resources.

We find that airport screening has only a moderate benefit during the early stages of the epidemic, as about 64% of infected individuals travel during the incubation period and exhibit symptoms an average of 3 d after arrival. Our estimate of duration between arrival and symptom onset is consistent with data of 20 imported cases reported to have arrived during the incubation period. Due to the logistical difficulty of identifying COVID-19 cases during the incubation period, some countries are requesting individuals to self-report any exposure to the virus, self-monitor for symptoms, and voluntarily quarantine if symptoms arise (17). Prior surveys of pandemic influenza suggest that the proportion of people self-isolating can range from 70 to 95% if those people are diagnosed with the disease (27, 28). The risk with this approach, however, is the uncertainty about people’s willingness to self-quarantine prior to symptom onset.

To curtail local outbreaks, it is important to identify imported cases in the incubation period before onward transmission occurs. We predicted that public health officials would have to identify and quarantine these imported cases within a week of arrival to limit the risk of transmission, with earlier detection of cases being more efficient. Early detection of cases could be critical, given recent studies estimating a shorter serial interval than previously reported, assuming transmission occurs during the asymptomatic period of infection (10, 29). As of February 21, there are at least 14 countries that have reported human-to-human transmission of SARS-CoV-2 (8). The US Center for Disease Control reported a case of human-to-human transmission on January 30, 2020, after the index patient was diagnosed with SARS-CoV-2 infection on January 21 (25). Our estimate of arrival time to the first transmission event corroborates with this report.

With the entire city of Wuhan under quarantine, stranded international travelers are being evacuated by their countries and quarantined for 2 wk, due to the rapidly growing epidemic (30–32). To curb the burden of the COVID-19 outbreak, efforts have mounted to rapidly develop an efficacious vaccine against SARS-CoV-2 (1, 33). Our study highlights the importance of complementary containment measures, such as contact tracing and voluntary quarantine, in limiting the global spread of the pandemic. With the availability of a SARS-CoV-2 vaccine, the risk of additional importation can be mitigated by focusing initial distribution of these vaccines in the regions where the imported cases have been identified and, secondarily, in regions that are highly connected to the affected areas.

## Methods

We used daily incidence data of COVID-19 outbreak within mainland China from December 8, 2019 to February 15, 2020, as well as airline network data, to predict the number of exported cases with and without measures of travel restriction and screening. Using a maximum likelihood approach, we calibrated the daily probability that an infected person would travel out of mainland China by fitting our predictions of exported cases to reported international incidence for cases that had a travel history to China (10, 34–39) (*SI Appendix*, Fig. S1). We assumed that infected individuals can travel over the entire course of their incubation or symptomatic period. The distribution of the incubation period was informed by clinical estimates that suggest an average duration of 5.2 d (*SI Appendix*, Table S1). The longest time window over which a symptomatic case could travel was informed by an empirical distribution of the duration between symptom onset and first medical visit (*SI Appendix*, Table S1). Using empirical distributions of these two durations, we evaluated the risk of an infected case being exported from the epicenter. To estimate the country-specific risk of importation of an individual infected with SARS-CoV-2, the probability of travel for an infected person was informed by country-specific weights that we calculated from flight data. These weights were proportional to the number of airports within the country that have direct flights to/from mainland China (40). We validated our country-specific estimates of importation risk using data from 21 countries reporting the arrival date of the first imported case (Table 1 and *SI Appendix*, Table S5).

For cases imported during the incubation period, we calculated likelihood distributions of the time between arrival and symptom onset using the distribution of the incubation period and probability of traveling over that period. We validated these estimates using publicly available reports (*SI Appendix*, Table S5). We then estimated the time to the first transmission event using the empirical estimates of serial internal distribution (time from symptom onset in the index patient to time of symptom onset in the secondary case) and distribution of incubation period (*SI Appendix*, Table S1).

We also evaluated the impact of other nonpharmaceutical interventions, including the effectiveness both of providing health questionnaires at the airport for self-identification of possible exposure in the last 14 d and of quarantining potentially exposed cases after contact tracing in mainland China. We calculated the impact of contact tracing in mainland China on the probability that an infected case in their incubation period would travel, based on the time interval from infection to quarantine.

### Data Availability.

The data and scripts used for the analysis can be found at https://github.com/WellsRC/Coronavirus-2019.

## Supplementary Material

Supplementary File
