# Supplementary material for: Impact of international travel and border control measures on the global spread of the novel 2019 coronavirus outbreak
Source: Proc Natl Acad Sci U S A. 2020 Mar 13;117(13):7504–9. doi: 10.1073/pnas.2002616117 (PMC7132249; doi:10.1073/pnas.2002616117)
Supplement: Supplementary File [file pnas.2002616117.sapp.pdf]

Supplementary Information for: Impact of international travel and border control measures on the global spread of the novel 2019 Coronavirus outbreak

Chad R. Wells, Pratha Sah, Seyed M. Moghadas, Abhishek Pandey, Affan Shoukat, Yaning Wang, Zheng Wang, Lauren A. Meyers, Burton H. Singer, and Alison P. Galvani

Epidemiological and clinical parameterization

We used early clinical estimates from the 2019 novel coronavirus (SARS-CoV-2) outbreak in Wuhan, China which specify the distribution of the incubation period to be log-normal with a mean of 5.2 days and a 95th percentile of 12.5 days (Table S1) (1). Uncertainty around the average duration of the incubation period was accounted for by fitting a gamma distribution to the 95% confidence interval of 4.1 days - 7 days and a mean of 5.2 days (1). The relationship between the uncertainty in the mean and uncertainty in the 95th percentile was not reported (1), we thus fixed the variance of the log-normal distribution. Assuming a maximum incubation period of 21 days, we generated a discrete version of this log-normal distribution, *f(x)*, and defined the probability distribution function as

$$f(x) = \begin{cases} \int_0^{0.5} h(s)\mathrm{d}s, & x = 0 \\ \int_{x-0.5}^{x+0.5} h(s)\mathrm{d}s, & x > 0 \end{cases}, \quad \text{(S1)}$$

where *h(s)* is the log-normal distribution. This distribution was normalized such that it summed to one (as 21 days is roughly the 99th percentile). We sampled individual incubation periods from the continuous log-normal distribution and then rounded the sampled number.

We used distributions of the time from symptom onset to first medical visit as well as to hospitalization of SARS-CoV-2 cases reported in the literature, as a proxy for the time in which a symptomatic individual will travel (Table S1)(1). Two time dependent distributions were obtained for each of these two duration - the first was based on cases who had symptom onset before January 1, and the second was for those who had symptom onset January 1 and after. For each distribution, we fit a Weibull distribution (1) and rounded the sampled duration to the nearest whole number.

Airline network weight

For the airline weight in our analysis, we utilized publicly available data of airline connections from 2014 (2). This weight was calculated as the number of airports within the country with direct flight connection to/from mainland China

$$\omega_i = \frac{n_i}{\sum_{c=1}^{N} n_c}, \quad \text{(S2)}$$

where n_i is the number of airports within the country *i* that have a direct flight connection to/from China. After the enforcement of travel lockdown on January 23, we removed the flights from Wuhan and recalculated this weight. Thus, we utilize all airline data in the weights for arrivals before January 23, 2020, and the adjusted weight for arrivals on January 23,2020 and after. We do not consider the number of airline routes in our baseline analysis, as these likely have changed since the last update of the dataset in 2014. However, the number of airports within the country that have a direct flight connection to/from mainland China should be relatively comparable.

Estimation of the probability of travel.

In our model fitting, we used daily incidence of COVID-19 from December 8, 2019 to February 15, 2020 (3–9). These cases were disaggregated into cases reported in Wuhan, Hubei, the rest of China, and internationally outside mainland China. For each infected case, we sampled an incubation period and the time from symptom onset to hospital admission from the maximum likelihood distributions. We first calculated the probability that infected individual *i* from the epicenter travels by plane over the course of the incubation period

$$Q_i = \sum_{s=t_I}^{t_S-1} \omega(s)p(s) \prod_{j=s}^{s-1}(1-p(j)), \tag{S3}$$

where t_S is the minimum of the time of symptom onset and travel restriction (for region appropriate case), $\omega(t)$ is the weight for all flights out of mainland China, and t_I is the time of infection (i.e. exposure). We do not account for the daily travel of an individual in their incubation period, as the data used for fitting is based on symptomatic individuals. We then calculated the daily probability of travel between symptom onset and first medical visit as:

$$W_{i,t} = \begin{cases} \omega(t)p(t)\prod_{s=t_s}^{t-1}(1-p(s)), & \text{for } t_s \leq t < t_M, \\ 0 & \text{otherwise} \end{cases} \tag{S4}$$

where t_m is the minimum of the time of first medical visit and travel ban. This equation expresses the probability that a symptomatic individual *i* travelled on day *t* and not before. Thus, we calculated the expected number of symptomatic cases for a given day as:

$$I_t = \sum_{i=1}^{N} T_{i,t}Q_i + W_{i,t}. \tag{S5}$$

where $T_{i,t}$ = 1 if the date of symptom onset for individual *i* is *t*, otherwise $T_{i,t}$ = 0. The number of cases travelling prior to their incubation period and then exhibiting symptoms after arrival is:

$$E_t = \sum_{i=1}^{N} T_{i,t}Q_i. \tag{S6}$$

We repeated this process 1,000 times and calculated the average trend.

We assumed that all reported infected cases acquired infection within mainland China, thus neglecting any reported cases of human-to-human transmission outside of China in the fitting. We estimated the probability of travel from the time of infection to the time of first medical visit, accounting for the travel lockdowns in Wuhan (January 23) and Hubei (January 25) and allowing for exportation from the rest of mainland China. To estimate the probability of travel per day during the course of the outbreak, we fitted the expected number of symptomatic cases outside of China (Eq S5) to the reported daily incidence outside of China and the number cases exhibiting symptoms after arrival (Eq S6) to incidence outside of China based on the time of symptom onset.

For a probability of travel per day in the calibrated range 0.003 to 0.03 (increasing at increments of 10^{-4}), we computed the log-likelihoods for the daily incidence outside of China using a Poisson distribution and the average trend in exported daily incidence. Based on arrival dates and dates of symptom onset of 30 cases, 20 exhibited symptoms after arrival (Table S5). Thus, we weighted the log-likelihood for the number cases exhibiting symptoms after arrival (Eq S6) by ~67% and the log-likelihood for the expected number of symptomatic cases outside of China (Eq S5) by ~33%. We determined the maximum likelihood estimate of the probability of travel per day and constructed a confidence interval using likelihood weighted sampling. Using this approach, we estimated that the probability of travel per day is 0.0068 (95% CI: 0.0059 - 0.0079). Assuming that individuals traveled up to the point of hospitalization, our estimate is 0.0060 (95% CI: 0.0052 - 0.0070).

Exportation probability and impact of border control measures

We assumed that no infected individuals travelled from Wuhan after the travel lockdown enforced on January 23, 2020(1, 10–14). Additional lockdowns in other cities in Hubei followed the one in Wuhan, with some reports indicating that public transportation was halted by 2:00 pm on January 24, 2020 (1, 10–14). As people potentially travelled out from these cities before 2:00pm on January 24, we restricted the travel for cases in Hubei, outside of Wuhan starting from January 25, 2020.

To estimate the exportation probability from mainland China, we used daily incidence of COVID-19 from December 8, 2019 to February 15, 2020, disaggregated into cases reported in Wuhan, Hubei, the rest of China, and internationally outside mainland China (3–9) . We sampled a time from symptom onset to first medical visit (after January 1) to generate a time of symptom onset for each SARS-CoV-2 case whose date of symptom onset was not specified.

For our baseline analysis, we sampled the incubation period and duration from symptom onset to first medical visit for each infected individual from the baseline distribution and fixed the probability of travel (see *Estimation of the probability of travel* section for further details). We calculated the daily probability that an infected case would be exported from mainland China. We make the simplifying assumption that the individual either travels or not (*e.g.* they cannot fly within

China the day before and then fly internationally). If the person does travel, we consider only the flights outside of China. For an infected case *i*, the probability of travel on day *t* was calculated as:

$$D_{i,t} = \begin{cases} \omega(t)p(t)\prod_{s=t_I}^{t-1}(1-p(s)), & t_I \leq t < t_S, \\ \omega(t)p(t)(1-p(t))^{t_S-t_I}\prod_{s=t_I}^{t-1}(1-p(s)), & t_S \leq t < t_M, \\ 0 & \text{otherwise} \end{cases}, \quad \text{(S7)}$$

where t_I is the time the individual *i* was infected, t_S is the time of symptom onset, t_M is the time in which the individual seeks medical attention, and $p(t)$ is the probability of traveling that day.

The expected number of cases exported outside of China for day *t* was calculated as:

$$F_t = \sum_{i=1}^{N} D_{i,t}, \quad \text{(S8)}$$

and the cumulative number of cases exported from China was calculated as:

$$C_t = \sum_{i=0}^{t} F_t. \quad \text{(S9)}$$

To estimate the number of individuals traveling during the incubation period at time *t* (G_t), we evaluated $D_{i,t}$ only for the time between exposure and symptom onset. Thus, the number of symptomatic people that traveled outside China was calculated as F_t - G_t, which provides us with an upper bound for the effectiveness of screening for symptoms at the airport.

We calculated the daily probability that at least one of these infected cases is exported as:

$$V_t = 1 - \prod_{i=1}^{N}(1 - D_{i,t}), \quad \text{(S10)}$$

and the probability that at least one case has been exported since the start of the outbreak was calculated as:

$$Z_t = 1 - \prod_{i=1}^{N}(1 - \sum_{j=0}^{t} D_{i,j}). \quad \text{(S11)}$$

Using the daily probability that at least one of these cases is exported, we estimated the expected time of the first exportation event as:

$$\mathbb{E}(t_E) = \sum_{j=0}^{\infty} j z(j), \tag{S12}$$

where

$$z(j) = Z_j - Z_{j-1}. \tag{S13}$$

We repeated this process 1,000 times and calculated the mean exportation probability under the baseline assumptions. We then repeated the entire process 1,000, bootstrapping the average duration of the incubation period, as well as the probability of travel per day, to calculate the 2.5 and 97.5 percentiles for our credible intervals.

Quarantine at the epicenter to curb exportation

We calculated the expected probability of exportation when individuals in the incubation period are quarantined after contact tracing. We estimate the expected probability that an infected case is exported as:

$$\mathbb{E}[\mathcal{P}|c] = \tilde{\omega} \sum_{i=0}^{21} f(i) \left(1 - (1 - \tilde{p})^{\min\{i,c\}}\right). \tag{S14}$$

where *c* is the time from infection to quarantine, $\tilde{\omega}$ is assumed to be the constant weight for flights out of mainland China, and $\tilde{p}$ is assumed to be a constant probability of travel per day (see *Estimation of the probability of travel* section for further details)

Identification of individuals travelling in their incubation period through health questionnaires .

To identify individuals travelling in their incubation period through health questionnaires , we examined the time since their last exposure, which we assumed to be the time of infection. For a given duration inquiry *q*, the probability of identifying an individual travelling in their incubation period is:

$$L(q) = \sum_{i=0}^{21} f(i) \left(\sum_{k=0}^{\min\{q,i-1\}} \frac{\tilde{\omega}\tilde{p}(1-\tilde{p})^k}{\tilde{\omega}\,(1-(1-\tilde{p})^i)} \right), \tag{S15}$$

where *i* is the duration of the incubation period , $\tilde{\omega}$ is assumed to be the constant weight for flights out of mainland China, and $\tilde{p}$ is assumed to be a constant probability of travel per day (see *Estimation of the probability of travel* section for further details).

Time to first infection after arrival in country

With no information of the generation time of the COVID-19, we used the serial interval (time from symptom onset in index patient to time in symptom onset in secondary case) as a proxy to estimate

the time to the first infection event. For the distribution of the serial interval, we used a negative binomial (a discrete analogues of the gamma distribution estimated in published work on COVID-19). For a given incubation duration *m* in the secondary case, we can evaluate the average time to the transmission event from the index case to the secondary case

$$\mathbb{E}(t_T|m) = \frac{\sum_{j=m}^{\infty}(j-m)b(j)}{1-B(m-1)}, \quad \text{(S16)}$$

where *b*(.) is the probability distribution function and *B*(.) is the cumulative probability distribution function for the serial interval. We then used these expected times and the distribution of the incubation period to provide an estimate for the average time from arrival to the first infection event.

Similarly, we calculated the expected time from arrival to symptom onset. For a given arrival time *k*, we calculated the average time from arrival to symptom onset by

$$\mathbb{E}(t_A) = \sum_{i=0}^{21} f(i) \left(\frac{\sum_{k=0}^{i-1}(i-k)\tilde{\omega}\tilde{p}(1-\tilde{p})^k}{\tilde{\omega}\,(1-(1-\tilde{p}))} \right) \quad \text{(S17)}$$

where *i* is the duration of the incubation period , $\tilde{\omega}$ is assumed to be the constant weight for flights out of mainland China, and $\tilde{p}$ is assumed to be a constant probability of travel per day (see *Estimation of the probability of travel* section for further details) .

To evaluate the uncertainty in the time from arrival to the first transmission event, we bootstrapped from the distributions of the time from arrival to symptom onset (Eq S17) and the time from symptom onset in the index case to the first transmission event (Eq S16).

Validation

We validated our estimates of the probability of travel further by examining this value based on the amount of data included in the fitting process (Figure S3). We obtained a comparable value for the probability of travel based on data of time of symptom onset after January 30, 2020, which is approximately 78% of the full dataset up to February 15, 2020.

We also examined the robustness of our estimates by evaluating the time of the first importation events using weights specified by the number of routes rather than the number of airports. We found that our likelihood estimate of the arrival time is robust to the change in the airline weight (Figure S4, Table S4).

Code availability

The excel file for Table S5, the code, and data used in the fitting and analysis can be found at https://github.com/WellsRC/Coronavirus-2019.

References

1. Q. Li, *et al.*, Early Transmission Dynamics in Wuhan, China, of Novel Coronavirus-Infected Pneumonia. *N. Engl. J. Med.* (2020) https:/doi.org/10.1056/NEJMoa2001316.

2. , OpenFlights: Airport and airline data (February 7, 2020).

3. Q. Li, *et al.*, Early Transmission Dynamics in Wuhan, China, of Novel Coronavirus-Infected Pneumonia. *N. Engl. J. Med.* (2020) https:/doi.org/10.1056/NEJMoa2001316.

4. , Wuhan Municipal Health and Family Planning Commission (February 8, 2020).

5. National Health Commision of the People's Republic of China, Xi Jinping made important instructions on the pneumonia epidemic of new coronavirus infection and emphasized that the safety of the people's lives and physical health should be the first to resolutely curb the spread of the epidemic, and Li Keqiang gave instructions. *National Health Commision of the People's Republic of China* (February 8, 2020).

6. National Health Commission of the People's Republic of China, Outbreak Report: National Health Commission of the People's Republic of China. *National Health Commission of the People's Republic of China* (February 8, 2020).

7. Hubei Provincial Health Committee, Hubei Provincial Health Committee. *Hubei Provincial Health Committee* (February 8, 2020).

8. , "Novel Coronavirus(2019-nCoV) Situation Report – 28" (World Health Organization, 2020) (February 25, 2020).

9. , China's Hubei reports 1,638 new confirmed cases of novel coronavirus infection - Xinhua | English.news.cn (February 13, 2020).

10. D. Cyranoski, A. Silver, Wuhan scientists: What it's like to be on lockdown. *Nature* (2020) https:/doi.org/10.1038/d41586-020-00191-5 (February 5, 2020).

11. , Weeks before lockdown, Wuhan authorities used "refrigerating strategy" to downplay coronavirus. *The Globe and Mail* (2020) (February 7, 2020).

12. W. M. /. William, Wuhan pneumonia virus continues to spread, Hubei orders 15 cities to be closed. *DW.COM* (February 7, 2020).

13. Y. Wang, China expands lockdowns to 25M people as coronavirus kills 25. *CTVNews* (2020) (February 5, 2020).

14. , Wuhan virus: China deploys army medics. *The Star Online* (2020) (February 7, 2020).

Table S1. Description of the distribution used in the analysis

Parameter	Distribution		Reference
Incubation period	Discrete log-normal (Truncated 21 days)	Mean: 5.2 days<br>Standard deviation: 3.91 days	(1)
Mean of incubation period	Gamma	Mean: 5.2 days<br>95% CI: 3.8 - 6.8 days	Fit to<br>95% CI: 4.1 - 7.0 days<br>(1)
Time from symptom onset to first medical visit (Onset before Jan. 1, 2020)	Discrete Weibull	Mean: 5.86 days<br>Standard deviation: 5.78 days	Fit to digitized distribution from (1)
Time from symptom onset to first medical visit (Onset Jan. 1, 2020 and after)	Discrete Weibull	Mean: 4.65 days<br>Standard deviation: 3.87 days	Fit to digitized distribution from (1)
Time from symptom onset to hospitalization (Onset before Jan. 1, 2020)	Discrete Weibull	Mean: 12.47 days<br>Standard deviation: 7.31 days	Fit to digitized distribution from (1)
Time from symptom onset to hospitalization (Onset Jan. 1, 2020 and after)	Discrete Weibull	Mean: 9.05 days<br>Standard deviation: 3.73 days	Fit to digitized distribution from (1)
Serial interval	Negative binomial	Mean: 7.5 days<br>Standard deviation: 3.4 days	(1)

Table S2. Estimates of the average probability of travel during various stages of infection

Time of travel	Average probability of travel
Epidemic: Travel during incubation period and travel lockdown (Calibration: Time to first medical visit)	0.2% (95% CI: 0.1% - 0.3%)
Epidemic: Travel during incubation period and no travel lockdown (Calibration: Time to first medical visit)	0.6% (95% CI: 0.4% - 0.9%)
Epidemic: Travel during incubation period plus time from symptom onset to first medical visit and travel lockdown	0.3% (95% CI: 0.3% - 0.4%)
Epidemic: Travel during incubation period plus time from symptom onset to first medical visit and no travel lockdown	1.1% (95% CI: 0.9% - 1.4%)
Epidemic: Travel during incubation period and travel lockdown (Calibration: Time to hospitalization)	0.2% (95% CI: 0.1% - 0.3%)
Epidemic: Travel during incubation period and no travel lockdown (Calibration: Time to hospitalization)	0.6% (95% CI: 0.4% - 0.8%)
Epidemic: Travel during incubation period plus time from symptom onset to hospitalization	0.4% (95% CI: 0.3% - 0.5%)
Epidemic: Travel during incubation period plus time from symptom onset to hospitalization and no travel lockdown	1.4% (95% CI: 1.2% - 1.7%)

Table S3. Estimates of the effectiveness of screening based on the calibration of the probability of travel

Measure	Calibration: Time to first medical visit	Calibration: Time to hospitalization
Cases exported (Travel lockdown)	230 (95% CI: 178 - 298)	264 (95% CI: 214 - 329)
Cases Exported (No Travel lockdown)	779 (95% CI: 632 - 967)	949 (95% CI: 794 - 1144)
Cases detected through screening (Travel lockdown)	82 (95% CI: 72 - 95)	133 (95% CI: 116 - 155)
Cases detected through screening (No travel lockdown)	351 (95% CI: 306 - 409)	570 (95% CI: 497 - 663)
Percentage of exported travel during incubation period (Travel lockdown)	64.3% (95% CI: 55.4% - 71.3%)	49.5% (95% CI: 40.4% - 57.4%)
Percentage of exported travel during incubation period (No travel lockdown)	54.9% (95% CI: 46.6% - 61.6%)	39.9% (95% CI: 32.2% - 46.6%)

Table S4. The estimate of the date of an infected case being exported to a given country in comparison to the first arrival of a reported infected case. The maximum likelihood estimate (MLE) is the date of the maximum likelihood from the calculated probability distribution. These estimates are calculated using airline weights based on the number of routes entering the country from China.

Country	Airline weight	Arrival date of first case	Estimated arrival date (MLE)	Estimated arrival date (Mean)	Standard deviation (days)
Australia	0.0025	January 6, 2020	January 19, 2020	January 17, 2020	7.6
Japan	0.0228	January 6, 2020	January 9, 2020	January 6, 2020	6.5
Thailand	0.00657	January 8, 2020	January 15, 2020	January 12, 2020	6.5
Nepal	0.000469	January 9, 2020	January 22, 2020	January 22, 2020	8.6
Taiwan	0.0258	January 12, 2020	January 8, 2020	January 5, 2020	6.6
India	0.00125	January 13, 2020	January 22, 2020	January 20, 2020	8.3
United States	0.00767	January 13, 2020	January 14, 2020	January 11, 2020	6.5
Vietnam	0.00235	January 13, 2020	January 20, 2020	January 18, 2020	7.7
United Arab Emirates	0.00203	January 16, 2020	January 20, 2020	January 18, 2020	7.9
Germany	0.00266	January 19, 2020	January 19, 2020	January 17, 2020	7.5
South Korea	0.0188	January 19, 2020	January 10, 2020	January 7, 2020	6.5
Sri Lanka	0.000156	January 19, 2020	January 22, 2020	January 23, 2020	8.7
Singapore	0.00579	January 20, 2020	January 16, 2020	January 13, 2020	6.6
Philippines	0.00297	January 21, 2020	January 19, 2020	January 16, 2020	7.4
Canada	0.00188	January 22, 2020	January 21, 2020	January 19, 2020	8
France	0.0011	January 22, 2020	January 22, 2020	January 21, 2020	8.4
Cambodia	0.00112	January 23, 2020	January 22, 2020	January 21, 2020	8.4
Finland	0.000642	January 23, 2020	January 22, 2020	January 22, 2020	8.6
Italy	0.00112	January 23, 2020	January 22, 2020	January 21, 2020	8.4
Sweden	0.000321	January 24, 2020	January 22, 2020	January 23, 2020	8.7
United Kingdom	0.00112	January 30, 2020	January 22, 2020	January 21, 2020	8.4

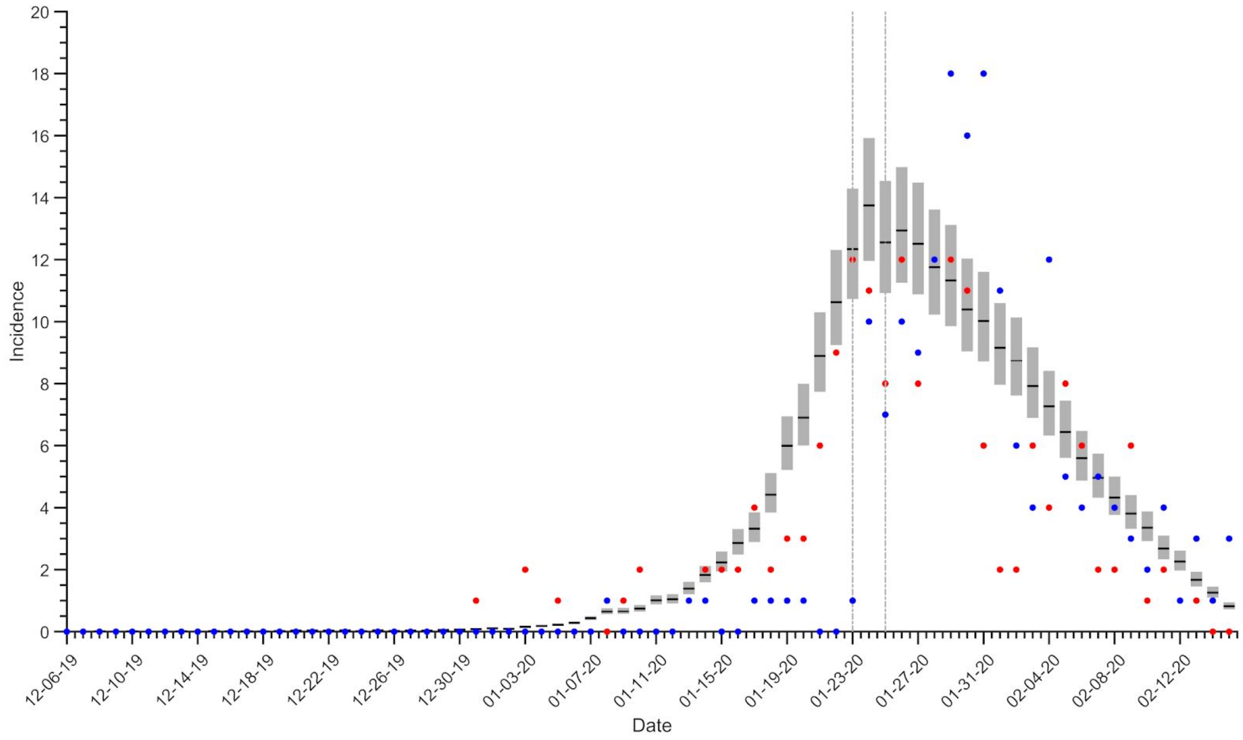

Figure S1. Model fit based on estimating the probability of travel per day. We fit the model (black line) to the incidence data based on the time of symptom onset (red dots) and time of the report (blue dots) by estimating the probability of travel per day, while accounting for the travel restrictions on January 23 in Wuhan and on January 25 for cities in Hubei province (gray vertical lines). The average incubation period used is 5.2 days and the time from symptom onset to first medical visit was used in the fitting. The gray area denotes the 95% credible interval. The weight for the log-likelihood of the time to symptom onset was 67% and the remaining 33% was for the log-likelihood of the time of reporting.

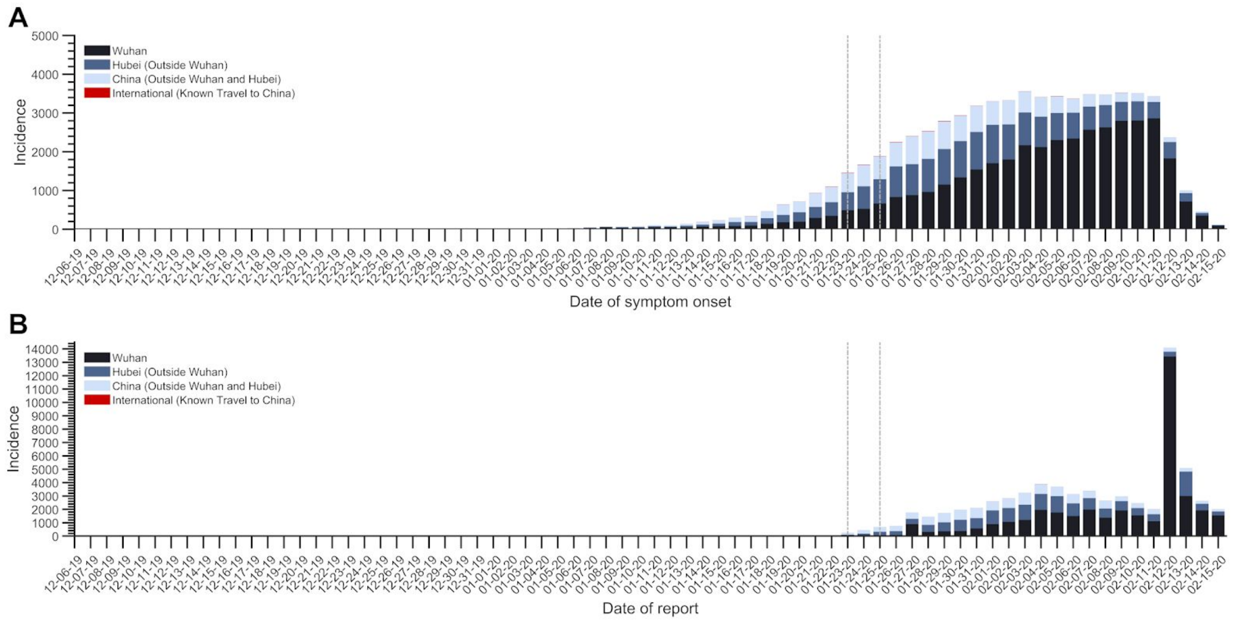

Figure S2. Data used in evaluating the risk of exportation from China. A) The incidence of cases in Wuhan (Dark blue), Hubie outside Wuhan (blue), and China outside Hubei, Wuhan (light blue), and international cases with reported travel to China (red) based on the date of symptom onset. For the cases with no known time of symptom onset and only reported, the time of symptom onset was estimated from sampled a time from symptom onset to first medical visit (after January 1). B) The reported cases used to supplement incidence of cases with an unknown time of symptom onset. The travel restrictions were enforced on January 23 in Wuhan and on January 25 for cities in Hubei province (gray vertical lines).

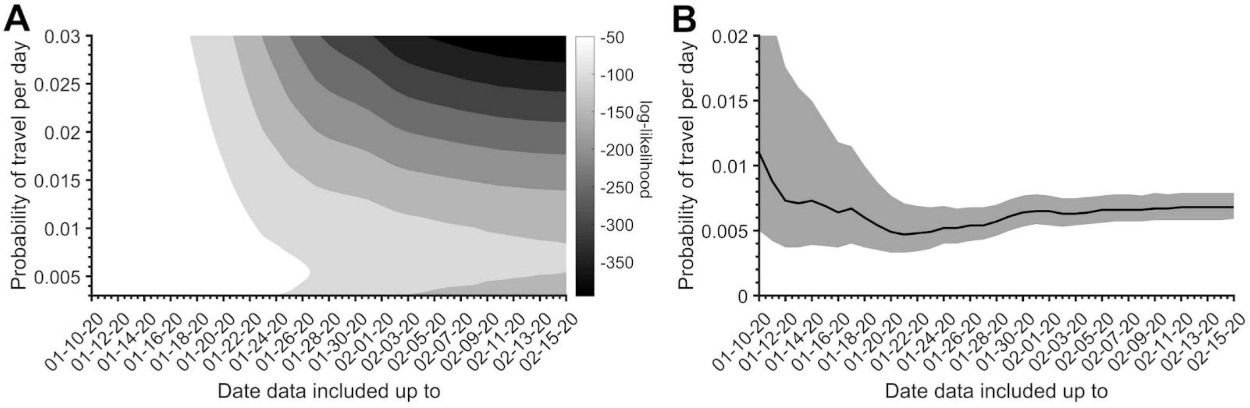

Figure S3. Validation of the estimate of the probability of travel. For a given date to which the model was informed to (x-axis), we examined the A) the likelihood profile for the probability of travel and B) the maximum likelihood estimate of the probability of travel (black line) and the corresponding 95% credible interval (gray area).

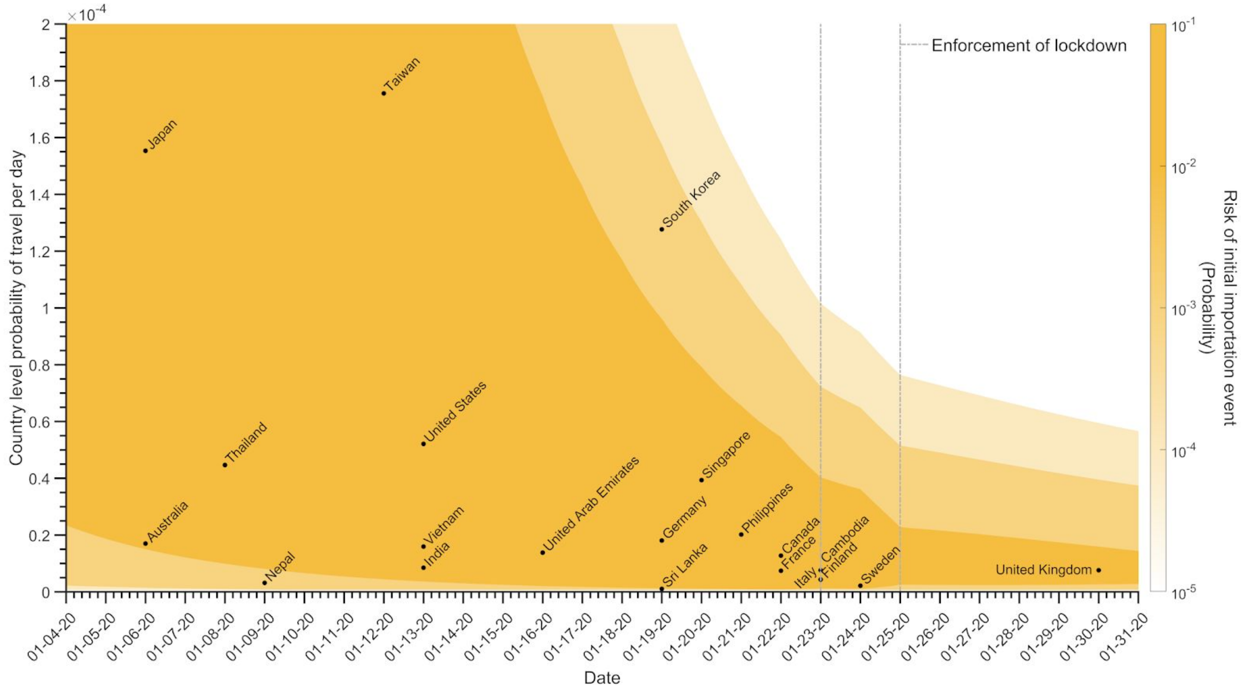

FigureS4: Country-level importation of SARS-CoV-2 cases. The risk of initial importation (gradient) based on probability of an individual in mainland China to travel by flight (y-axis), estimated each day between January 4, 2020 to January 31,2020 (x-axis). The country level weights are based on the number of routes from China to the country. The correlation was not significant between the reported arrival date of the initial case in 21 countries/regions and the airline weight based on the number of routes (r = -0.42, p-*value* =0.06). Vertical dashed lines indicate the travel bans that were enforced on January 23 in Wuhan and on January 25 for other cities in Hubei province.

Arrived symptomatic
Arrived asymptomatic
Time symptom onset uncertain

Country	Arrival date	Date of symptom onset	Confirmation date	Arrival date to date of symptom onset	Notes	Reference (Date range links accessed: January 31, 2020 to Febraury 8, 2020)
Australia	January 6, 2020				Unwell shortly after the flight	https://www.sbs.com.au/news/calm-urged-as-china-s-coronavirus-reaches-australia-with-four-confirmed-cases, https://www.abc.net.au/news/2020-01-25/first-confirmed-coronavirus-case-australian-as-china-toll-rises/11900428
Australia	January 9, 2020				Unwell shortly after the flight	https://www.sbs.com.au/news/calm-urged-as-china-s-coronavirus-reaches-australia-with-four-confirmed-cases
Australia	January 19, 2020	January 23, 2020		4	Arrived with no symptoms and went to see GP once they started to develop symptoms	https://www.sbs.com.au/news/calm-urged-as-china-s-coronavirus-reaches-australia-with-four-confirmed-cases, https://www.abc.net.au/news/2020-01-25/first-confirmed-coronavirus-case-australian-as-china-toll-rises/11900428
Cambodia	January 23, 2020	January 25, 2020			60-year-old Chinese male	https://www.voanews.com/science-health/coronavirus-outbreak/cambodia-confirms-first-coronavirus-case
Canada	January 22, 2020		January 25, 2020		Reported to be aymptomatic at time of testing (wife of confirmed case), no indication if or when symptoms appeared	https://toronto.ctvnews.ca/second-case-of-coronavirus-in-toronto-confirmed-by-federal-health-officials-1.4788223
Canada	January 22, 2020	January 22, 2020	January 23, 2020	0	Had minor symptoms on flight and hospitalized shortly after	https://news.ontario.ca/mohltc/en/2020/01/ontario-confirms-first-case-of-wuhan-novel-coronavirus.html
Canada	January 23, 2020	January 24, 2020		1	Student at Western University. Self-quaratined and exhibited mild symptoms and weakly positive	https://nationalpost.com/news/world/coronavirus-live-updates-wuhan-virus-china-2019ncov
Canada				1	Reprorted having symptoms 24 hrs after flight	https://vancouversun.com/news/local-news/five-things-to-know-about-coronavirus-in-b-c
Finland	January 23, 2020	January 26, 2020		3	32 yr old woamn from Wuhan. Flew to Helsinki Airport and took a connecting flight to Ivalo Airport in Inari (potentially exposed 15 people)	https://www.helsinkitimes.fi/finland/finland-news/domestic/17272-finland-s-first-coronavirus-patient-began-to-show-symptoms-three-days-ago.html
France	January 22, 2020	January 23, 2020		1	Traveled to France Wed and Thurs reported symptoms	http://www.rfi.fr/en/international/20200125-french-health-authorities-confirm-three-cases-new-coronavirus-first-europe, https://www.aljazeera.com/news/2020/01/france-confirms-european-coronavirus-cases-200124195752666.html
Germany	January 19, 2020				Arrived in Germany on the 19th , unclear when symptoms of 2019-nCoV developed	https://www.thelocal.de/20200128/first-coronavirus-case-confirmed-in-germany-bavarian-health-ministry, https://www.nejm.org/doi/full/10.1056/NEJMc2001468, https://www.sciencemag.org/news/2020/02/paper-non-symptomatic-patient-transmitting-coronavirus-wrong
India	January 13, 2020				Arrived in Ujjain on January 13 from Hubei province. Was kept in quaratine then moved to isolation.	https://www.khaleejtimes.com/international/india/indian-student-from-wuhan-returns-home-hospitalised-with-coronavirus-like-symptoms
India	January 29, 2020				Student from Wuhan University in China (Report indicates posibly one of thos 173 that returned on Wed. Jan 29)	https://fusion.werindia.com/hot-from-the-oven/coronavirus-confirmed-in-kerala
Italy	January 23, 2020				Husband (67) and wife (66) arrived in Italy. No indication about when symptoms arose	https://www.wantedinmilan.com/news/coronavirus-italy-declares-state-of-emergency.html
Japan	January 6, 2020	January 3, 2020	January 15, 2020	-3	30 years old man, Jan-3 fever, Jan-6 arrived Japan and checked with Doctor for fever, Jan-10 in hospital, Jan-15 recovered and confirmed as the very first coronvirus case in Japan	https://www.mhlw.go.jp/english/,http://www.chinacdc.cn/en/,https://shimo.im/sheets/tyWrrrqppYVwQtCW/oURp4/?from=singlemessage&isappinstalled=0
Japan	January 13, 2020		January 30, 2020		50 years old man, visited Wuhan from Dec-24 to Jan-13, confirmed with coronvirus on Jan-30	https://www.mhlw.go.jp/english/,http://www.chinacdc.cn/en/,https://shimo.im/sheets/tyWrrrqppYVwQtCW/oURp4/?from=singlemessage&isappinstalled=0
Japan	January 18, 2020		January 25, 2020		30 years old woman, Jan-18 from Wuhan to Japan, Jan-25 confirmed	https://www.mhlw.go.jp/english/,http://www.chinacdc.cn/en/,https://shimo.im/sheets/tyWrrrqppYVwQtCW/oURp4/?from=singlemessage&isappinstalled=0
Japan	January 19, 2020	January 19, 2020	January 24, 2020	0	40 years old man, Jan-19 arrived from Wuhan, fever, Jan-24 confirmed	https://www.mhlw.go.jp/english/,http://www.chinacdc.cn/en/,https://shimo.im/sheets/tyWrrrqppYVwQtCW/oURp4/?from=singlemessage&isappinstalled=0
Japan	January 21, 2020	January 22, 2020	January 28, 2020	1	40 years old woman, Jan-21 arrived from Wuhan, Jan-22 show symptoms, Jan-28 confirmed	https://www.mhlw.go.jp/english/,http://www.chinacdc.cn/en/,https://shimo.im/sheets/tyWrrrqppYVwQtCW/oURp4/?from=singlemessage&isappinstalled=0
Japan	January 22, 2020	January 23, 2020	January 30, 2020	1	20 years old woman, visited Wuhan Jan-16 to Jan-22, arrived on Jan-22, showed cough symptoms Jan-23, went to hospital for fever Jan-28, confirmed on Jan-30	https://www.mhlw.go.jp/english/,http://www.chinacdc.cn/en/,https://shimo.im/sheets/tyWrrrqppYVwQtCW/oURp4/?from=singlemessage&isappinstalled=0
Maylasia	January 23, 2020	January 29, 2020		6	This case was supposabley infected in Singapore (not used in the importation analysis since not travel to China)	https://www.channelnewsasia.com/news/asia/wuhan-coronavirus-malaysia-first-citizen-case-malaysian-12389728
Nepal	January 9, 2020			4	32 year old student arrived from Wuhan, came to hosptial 4 days later with fever, cough and shortness of breath. No indication of status on arrival	https://www.washingtonpost.com/world/coronavirus-china-live-updates/2020/01/24/4e678f9c-3e03-11ea-afe2-090eb37b60b1_story.html
Philippines	January 21, 2020			4	Addmitted to hospital with cough four days after arrival, with no later symptoms	https://www.cnbc.com/2020/01/30/india-confirms-first-case-of-the-coronavirus.html
Russia			January 31, 2020		On Firday (Jan 31) Russia confirmed first two cases (Unknown as to how long they had been in Russia)	https://www.wabcradio.com/2020/02/02/the-latest-coronavirus-info-3rd-person-being-tested-in-nyc-world-health-organization-is-working-with-google-to-control-rumors-and-misinformation-about-the-virus/,https://www.who.int/docs/default-source/coronaviruse/situation-reports/20200201-sitrep-12-ncov.pdf?sfvrsn=273c5d35_2
Singapore	January 20, 2020	January 20, 2020		0	Had sore throat on flight and had fever next day	https://www.channelnewsasia.com/news/singapore/wuhan-virus-pneumonia-singapore-confirms-first-case-12312860
South Korea	January 19, 2020	January 19, 2020	January 20, 2020	0	a 35 years old women, arrived on Jan-19 with fever symptom, Jan-20 confirmed	https://www.who.int/csr/don/21-january-2020-novel-coronavirus-republic-of-korea-ex-china/en/,http://www.chinacdc.cn/en/,https://shimo.im/sheets/tyWrrrqppYVwQtCW/oURp4/?from=singlemessage&isappinstalled=0
South Korea	January 20, 2020	January 20, 2020	January 26, 2020	0	54 old man, Jan-20 arrived, showed minor symptom, 25 reported to CDC, Jan-26 confirmed.	https://www.cdc.go.kr/cdc_eng/,http://www.chinacdc.cn/en/,https://shimo.im/sheets/tyWrrrqppYVwQtCW/oURp4/?from=singlemessage&isappinstalled=0
South Korea	January 20, 2020	January 21, 2020	January 26, 2020	1	55 years old man, visited Wuhan, arrived Jan-20, Jan-21 visited hospital for fever, Jan-25 additional symptoms, Jan-26 confirmed	https://www.cdc.go.kr/cdc_eng/,http://www.chinacdc.cn/en/,https://shimo.im/sheets/tyWrrrqppYVwQtCW/oURp4/?from=singlemessage&isappinstalled=0
South Korea	January 20, 2020	January 20, 2020	January 26, 2020	0	54 old man, Jan-20 arrived, showed minor symptom, 25 reported to CDC, Jan-26 confirmed.	https://www.cdc.go.kr/cdc_eng/,http://www.chinacdc.cn/en/,https://shimo.im/sheets/tyWrrrqppYVwQtCW/oURp4/?from=singlemessage&isappinstalled=0
South Korea	January 22, 2020	January 22, 2020	January 22, 2020	0	a 50 some old man, 1.10 show fever symptom, 22 arrived Korea and confirmed with coronvirus.	https://www.cdc.go.kr/cdc_eng/,http://www.chinacdc.cn/en/,https://shimo.im/sheets/tyWrrrqppYVwQtCW/oURp4/?from=singlemessage&isappinstalled=0
Sri Lanka	January 19, 2020	January 25, 2020		6	Chinese woman in her 40's	https://nationalpost.com/pmn/health-pmn/sri-lanka-confirms-first-case-of-coronavirus-health-official
Sweden	January 24, 2020				Woman in 20's had visited Wuhan. Reported had no symptoms upon arrival . No duration between arrival and symptom onset. Since there was no date of symptom onset specified, we did not include in symptomatic arrival analysis	https://www.thelocal.se/20200131/first-case-of-coronavirus-confirmed-in-jonkoping-sweden
Taiwan	January 12, 2020	January 21, 2020	January 30, 2020	9	A man in his 40s, worked in Wuhan since Sept. 2019, returned to Taiwan on Jan 12, showed symptom on Jan. 21, diagnosed as common fever on Jan. 22, confirmed with coronvirus on Jan. 30.	https://www.cdc.gov.tw/En,http://www.chinacdc.cn/en/,https://shimo.im/sheets/tyWrrrqppYVwQtCW/oURp4/?from=singlemessage&isappinstalled=0
Taiwan	January 20, 2020	January 26, 2020	January 28, 2020	6	A woman in her 50s, worked in Wuhan since Oct, 2019, arrived Taiwan on Jan. 20, fever and muscle pain on Jan. 25, confirmed on Jan 27; Her husband showed symptoms on Jan. 26, confirmed on Jan. 28.	https://www.cdc.gov.tw/En,http://www.chinacdc.cn/en/,https://shimo.im/sheets/tyWrrrqppYVwQtCW/oURp4/?from=singlemessage&isappinstalled=0
Taiwan	January 20, 2020	January 25, 2020	January 27, 2020	5	A woman in her 50s, worked in Wuhan since Oct, 2019, arrived Taiwan on Jan. 20, fever and muscle pain on Jan. 25, confirmed on Jan 27; Her husband showed symptoms on Jan. 26, confirmed on Jan. 28.	https://www.cdc.gov.tw/En,http://www.chinacdc.cn/en/,https://shimo.im/sheets/tyWrrrqppYVwQtCW/oURp4/?from=singlemessage&isappinstalled=0
Taiwan	January 21, 2020	January 23, 2020	January 24, 2020	2	Person traveled from Wuhan to Taiwan on Jan. 21, doctor visit on Jan 23, confirmed on Jan. 24	https://www.cdc.gov.tw/En,http://www.chinacdc.cn/en/,https://shimo.im/sheets/tyWrrrqppYVwQtCW/oURp4/?from=singlemessage&isappinstalled=0
Taiwan	January 22, 2020	January 25, 2020	January 28, 2020	3	Two women in their 70s living in Wuhan, arrived Taiwan on Jan. 22, showed symptoms on Jan. 25, confirmed on Jan. 28;	https://www.cdc.gov.tw/En,http://www.chinacdc.cn/en/,https://shimo.im/sheets/tyWrrrqppYVwQtCW/oURp4/?from=singlemessage&isappinstalled=0
Taiwan	January 22, 2020	January 25, 2020	January 28, 2020	3	Second woman who arrived on Jan 22.	https://www.cdc.gov.tw/En,http://www.chinacdc.cn/en/,https://shimo.im/sheets/tyWrrrqppYVwQtCW/oURp4/?from=singlemessage&isappinstalled=0
Taiwan	January 25, 2020	January 22, 2020	January 26, 2020	-3	A woman in her 50s, visited Wuhan Jan. 13-15, then traveled in Europe from Jan 16-25, cough start Jan. 22 and become worse on Jan. 25, Jan-25 arrived Taiwan and report to hospital and confirmed on Jan. 26;	https://www.cdc.gov.tw/En,http://www.chinacdc.cn/en/,https://shimo.im/sheets/tyWrrrqppYVwQtCW/oURp4/?from=singlemessage&isappinstalled=0
Thailand	January 8, 2020	January 5, 2020		-3	61 year old woman from Wuhan travelled with five family memembers in a tour group of 16.	https://www.who.int/csr/don/14-january-2020-novel-coronavirus-thailand-ex-china/en/
UAE	January 16, 2020		January 23, 2020		Grandmother fell ill and went to seek medical help on the Janaury 23, but unclear as to when symptoms first appeared	https://www.cnbc.com/2020/01/29/first-middle-east-cases-of-coronavirus-confirmed-in-the-uae.html
UK	January 30, 2020				Reports indicate that the individual arrive a few days prior to symptoms. We conservatiely assumed they arrived one day prior to confirmation.	https://metro.co.uk/2020/01/31/coronavirus-arrives-uk-two-people-test-positive-deadly-disease-12136884/
USA	January 13, 2020				Began to feel unwell a few days after arrival, but no clear indication as to when symptom onset occurred (i.e. sore throat or cough)	https://edition.cnn.com/asia/live-news/coronavirus-outbreak-hnk-intl-01-24-20/index.html
USA	January 15, 2020	January 19, 2020		4	Male in 30's recently returned from Wuhan. Seeked care when became ill four days after arriving in the United States	https://www.cnn.com/2020/01/21/health/wuhan-coronavirus-first-us-case-cdc-bn/index.html
Vietnam	January 13, 2020	January 17, 2020		4	66 year old male experienced fever Janaury 17, son (who man met upon arrival) exhibit similar symoms on the 20th	https://thehill.com/policy/healthcare/public-global-health/479542-vietnam-reports-first-coronavirus-cases
